# Serum NGAL and copeptin levels as predictors of acute kidney injury in asphyxiated neonates

**DOI:** 10.1007/s10157-016-1320-6

**Published:** 2016-09-02

**Authors:** Małgorzata Baumert, Piotr Surmiak, Andrzej Więcek, Zofia Walencka

**Affiliations:** 1 Department of Neonatology, School of Medicine in Katowice, Medical University of Silesia, Medyków 14 Street, 40-752 Katowice, Poland; 2 Department of Nephrology, Endocrinology and Metabolic Diseases, School of Medicine in Katowice, Medical University of Silesia, Francuska 20/24 Street, 40-027 Katowice, Poland

**Keywords:** Asphyxia, Acute kidney injury, LCN2 protein, Copeptins, Osmolality

## Abstract

**Background:**

Acute kidney injury (AKI) is the most common complication of perinatal asphyxia. Recent research indicates that serum neutrophil gelatinase-associated lipocalin (NGAL) is an early marker for AKI, but there are the lacks of data about its use in term neonates with perinatal asphyxia.

**Methods:**

A prospective cohort study was conducted on 43 term neonates. Umbilical cord blood and 24 h after birth serum NGAL, copeptin, creatinine, and molality were measured in all asphyxiated and controls neonates.

**Results:**

During the study period, 8 of asphyxiated nenates (18.6 %) suffered from AKI, while 35 newborns have no signs of AKI and 30 healthy infants. We did not observe any differences in creatinine and copeptin levels, as well as serum osmolality in all three investigated groups (AKI, no-AKI, and controls) in cord blood, and 24 h after birth. Serum NGAL levels in umbilical cord blood were significantly higher in the AKI group (174.3 ng/mL) compared with no-AKI (88.5 ng/mL, *p* = 0.01) and control groups (28.5 ng/mL, *p* < 0.001), and 24 h after birth (respectively, AKI 152.5 ng/mL vs no-AKI 74.9 ng/mL, *p* = 0.02 vs controls 39.1 ng/mL, *p* < 0.001). NGAL concentration showed a strong negative correlation to umbilical artery pH (Rho = −0.42, *p* = 0.04), base excess (Rho = −0.31, *p* = 0.03), and Apgar score in 1st min (Rho = −0.41, *p* = 0.02) and 5th min of life (Rho = −0.20, *p* = 0.001). ROC curve analysis demonstrated a good predictive value for NGAL levels (>140.7 ng/mL) which allows to diagnose AKI in asphyxiated patients with 88.9 % sensitivity (95 % CI 75–95 %) and 95.0 % specificity (95 % CI 76–99 %).

**Conclusion:**

NGAL seems to be a promising marker, even in subclinical AKI in neonates, due to its high specificity, but copeptin did not meet expectations.

## Introduction

Acute kidney injury (AKI) is a complex disorder with clinical manifestations ranging from mild dysfunction to complete anuric kidney failure. Renal failure is characterized by increased serum levels of creatinine and nitrogenous waste products, decreased in glomerular filtration rate and imbalance in water and electrolyte homeostasis [1]. Diagnosis of AKI in neonates is difficult as many of them have non-oliguric renal failure, especially in premature infants [2]. Moreover, serum creatinine concentration during the first few days after birth reflects the mother’s and not the infant’s kidney function [3].

There are many reasons for development of AKI in newborns. Renal failure may have a prenatal onset in congenital disease, especially in genetic diseases, and in the postnatal period maybe related to hypoxic ischemic injury, intubation at birth, respiratory distress syndrome, toxic insults, etc. [1, 4]. Some authors reported the high occurence of AKI in neonates with perinatal asphyxia [5, 6]. In a study by Abu-Haweleh, perinatal asphyxia, with a prevalence of 42 %, was the most common predisposing factor for AKI in neonates and thus, associated with high mortality [7]. As neonates, with AKI, are at risk for developing chronic kidney disease and hypertension in the adult life, we search for novel markers that can improve the diagnosis of AKI within the first hours of an insult allowing the implementation of an effective prophylactic actions and/or treatment.

One of these novel biomarkers of AKI is neutrophil gelatinase-associated lipocalin (NGAL). It is known that serum and urinary levels of NGAL are elevated in neonates with AKI after cardiac surgery [8], and in a critically ill pediatric population [9].

In our previous studies, we demonstrated that NGAL can be a valuable biomarker of acute incidence of perinatal asphyxia in neonates [10, 11]. Hence, we want to test our hypothesis by demonstrating that elevated serum NGAL concentration is also an invaluable marker of AKI resulting from hypoxia.Moreover, we would also like to determine whether copeptin plays an important role in the evaluation of AKI. Copeptin is released in an equimolar ratio to vasopressin (AVP); however, it is more stable in circulation and easy to measure. Serum copeptin levels have been found to closely resemble the production of AVP, which is strongly related to serum osmolality [12]. Serum copeptin levels are increased in response to increased osmolality and dehydratation [13]. Some authors noticed that serum copeptin concentration was strongly related to factors associated with perinatal stress, such as asphyxia [14].

## Material

Among 1,673 infants, born at the University Hospital in Katowice, Poland between January 2012 to May 2014, 43 term neonates were enrolled in a prospective study, after obtaining informed written consent from their parents. This study was approved by the Human Ethics Committee of the Medical University of Silesia. The collection of umbilical cord blood and blood samples 24 h after birth was required for this research.

Indicators of acute perinatal asphyxia included: the presence of a hypoxic event immediately prior or during delivery, history of fetal distress (bradycardia, late decelerations), metabolic acidosis (base deficit ≥−16 mmol/l) in the arterial umbilical cord blood, and pH ≤7.0.

Categorization of the enrolled asphyxiated neonates into asphyxia subgroups (AKI, no-AKI) was based on the Acute Kidney Injury Network (AKIN) criteria, as persistently increased serum creatinine (>1.5 mg/dL) for at least 24 h or rising values >0.3 mg/dL from the baseline [15].

Thirty apparently healthy neonates of comparable gestational age born after an uncomplicated pregnancy and labor comprised the control group.

In our study, we excluded preterm neonates, with congenital abnormalities or chromosomal anomalies, newborns of mothers suffering from diabetes mellitus, hypertension, pre-eclampsia, and children of multiple pregnancies and metabolic disorders, and with evidence of congenital infections, as well as those that were born to mothers with clinical chorioamnionitis.

Patient care and monitoring were performed as part of the hospital’s standard protocol. Neonates after the incidence of perinatal asphyxia were transferred to NICU, and acid–base balance was obtained 1 h after delivery. Needs for institution of hypothermia protocol was determined according to the recommendations from “Total Body Hypothermia for Perinatal Asphyxia (TOBY)” study group [16]; however, none of neonates from asphyxiated group met the criteria for whole body cooling.

## Methods

Blood samples were drawn immediately after delivery of the child from the umbilical artery from the placental side of the cord and at 24 h after birth from the peripheral neonatal vessels. Blood samples were used for measurement of acid–base balance and serum NGAL, copeptin, creatinine, lactate levels as well as serum osmolality.After centrifugation, cord blood serum was frozen in tubes at −80 °C. Serum copeptin concentration was determined using the immunoluminometric assay (Brahms CT-pro AVP LIA, Brahms GmbH, Hennigsdorf, Germany). The detection limit of the assay was 0.4 pmol/L.

Serum NGAL concentration was determined using the sandwich enzyme immunoassay for the quantitative measurement of human lipocalin-2 (BioVendor- Labolatorni medicina a.s. Brno, Czech Republic). The detection limit of the assay was 0.02 ng/mL. Blood gas analyses were established using a model Rapidlab 865 Blood Gas Analyzer (Siemens Medical Solutions Diagnostics, Bad Nauheim, Germany). Serum creatinine concentration was measured by a kinetic colorimetric Jaffe method (Modular P Analyzer; Roche Almere, the Netherlands). Serum osmolality was determined using a cryoscopic method establishing its freezing temperature with the use of the Vapor Pressure Osmometer 5520.

### Statistical analysis

Continuous variables among the groups were compared using the Kruskal–Wallis and Mann–Whitney *U* tests, while categorical variables were compared using Fisher’s exact or Levene’s tests. Quantitative variables were presented as mean or median and 95 % confidence intervals, whereas qualitative variables were shown as percentages. Spearman correlation coefficients were calculated between NGAL, copeptin levels, and clinical variables. We also performed ROC curve analysis to determine the cutoff values of biochemical parameters as diagnostic markers of acute kidney injury in asphyxiated neonates. Statistical analysis was performed using standard procedures available in STATISTICA 10 (Statsoft Polska Inc.) and MedCalc Software Version 12.7.4. Statistical inferences were based on the level of significance *p* < 0.05.

## Result

During the study period, 43 neonates were born asphyxiated-8 of them (18.6 %) developed AKI stage 1 based on serum creatinine AKIN criteria, while 35 newborns did not meet diagnostic criteria for AKI. Renal replacement therapy was not required. During the hospitalization, we observed in neonates with AKI creatinine levels normalization average on 3rd day of life.

The demographic and clinical characteristics of the studied neonates are listed in Table 1. There were no significant differences in these groups, except for the Apgar score (*p* < 0.001).

**Table 1:** Demographic and perinatal characteristics of asphyxiated and control neonates

Variable	Asphyxiated neonates (*n* = 43)	Control group (*n* = 30)	*p* value
Mother’s age (years)	27 (25–31)	28 (24–31)	0.48
Cesarean section (%)	85	71	0.18
Gender male/female (%)	54/46	61/39	0.58
Weeks of gestation (weeks)	37 (36–37)	38 (37–39)	0.11
Apgar 1st min (pts)	4 (3–6)	8 (8, 9)	<0.001
Apgar 5th min (pts)	6 (5–7)	9 (8, 9)	<0.001

Results are presented as medians and (95 % confidence intervals) or percentages. *p* value from Kruskal–Wallis or Fisher’s exact tests

There were also no significant differences in serum copeptin levels and serum osmolality in all three groups (AKI, no-AKI, and controls) in cord blood, and 24 h after birth, as shown in Table 2. There were also no differences in serum creatinine in umbilical cord blood and after 24 h in our population arranged by gestational age and birth weight, shown in Table 3.

**Table 2:** Serum levels of creatinine (mg/dL), copeptin (pg/mL), NGAL (ng/mL), and serum osmolality (mmol/kg H_2_O) in cord blood and in serum after 24 h of life in asphyxiated (AKI and no-AKI) newborns and in the control group

	Asphyxiated *n* = 43	Controls *n* = 30	*p* value	AKI *n* = 8	No-AKI *n* = 35	*p* value
Umbilical creatinine (mg/dL)	0.8 (0.7–0.8)	0.9 (0.8–1.0)	0.56	0.9 (0.7–1.1)	0.8 (0.8–0.9)	0.26
Serum creatinine after 24 h (mg/dL)	0.8 (0.8–0.9)	0.9 (0.8–1.0)	0.62	1.0 (0.8–1.2)	0.8 (0.8–0.9)	0.09
Umbilical NGAL (ng/mL)	146.4 (105.8–187.0)	28.5 (21.9–35.0)	<0.001	174.3 (117.8–230.7)	88.5 (59.1–118.0)	<0.001
Serum NGAL after 24 h (ng/mL)	113.5 (85.6–141.4)	39.1 (25.2–53.1)	0.06	152.5 (80.1–224.8)	74.9 (55.5–94.4)	<0.001
Umbilical copeptin (pg/mL)	512.9 (429.2–596.6)	566.7 (452.4–681.1)	0.78	660.1 (273.5–1026.6)	520.8 (456.3–585.4)	0.32
Serum copeptin after 24 h (pg/mL)	468.6 (396.7–540.5)	429.1 (342.8–515.5)	0.41	439.9 (310.3–569.5)	455.1 (394.8–515.3)	0.51
Umbilical serum osmolality (mmol/kg H_2_O)	284.5 (282.9–285.1)	284.1 (281.7–286.5)	0.72	286.4 (282.8–289.9)	284.1 (282.7–285.5)	0.29
Serum osmolality after 24 h (mmol/kg H_2_O)	283.8 (281.9–285.0)	284.0 (282.3–285.0)	0.35	283.1 (280.6–285.9)	284.1 (282.8–285.4)	0.76

Results are shown as medians and (95 % confidence intervals). *p* value from Kruskal–Wallis and Levene’s tests

*NGAL* neutrophil gelatinase-associated lipocalin, *AKI* acute kidney injury

**Table 3:** Serum creatinine values in umbilical cord blood and after 24 h in our population arranged by gestational age and birth weight

Variable	Gestational age		*p* value	Birth weight		*p* value
	37–38	39–40		<2500 g	>2500 g	
Creatinine level (mg/dL)						
Umbilical cord blood	0.74 (0.48–1.10)	0.68 (0.37–1.05)	0.41	0.81 (0.56–1.21)	0.65 (0.34–1.18)	0.18
24 h after	0.68 (0.36–1.25)	0.75 (0.44–1.15)	0.36	0.78 (0.39–1.09)	0.72 (0.42–1.27)	0.35

Results are presented as median values and (minimum and maximum values). *p* value from Mann–Whitney *U* test

**Fig. 1:**
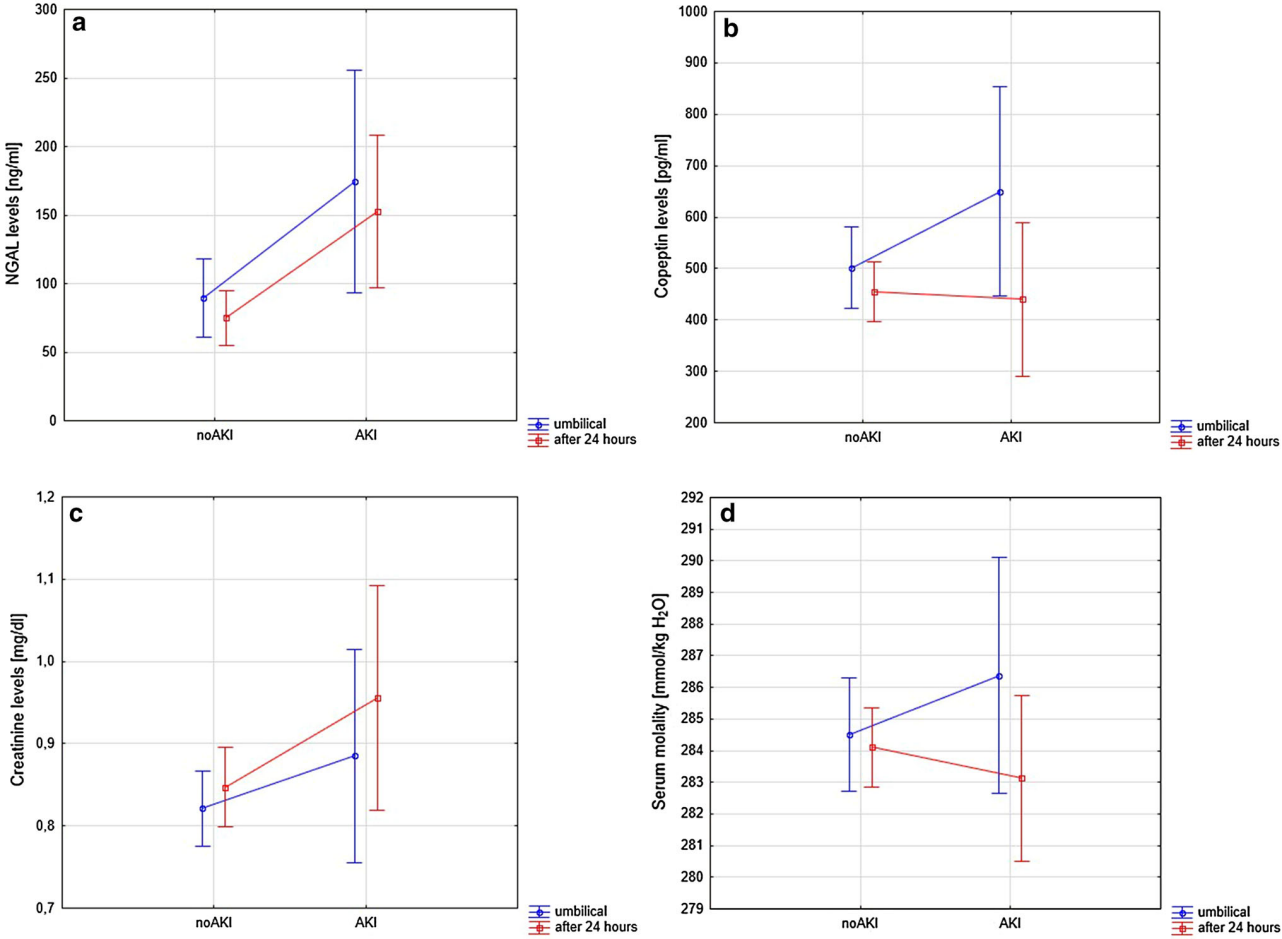
NGAL, copeptin and creatinine levels, and serum osmolality in neonates with acute kidney injury (AKI) and newborns no-AKI in umbilical cord blood and 24 h after birth. Results are shown as medians and 95 % confidence intervals. *NGAL* neutrophil gelatinase-associated lipocalin, *AKI* acute kidney injury

Although median serum copeptin concentrations were elevated within the AKI group (660.1 pg/mL) as compared with no-AKI (520.8 pg/mL) and controls (566.7 pg/mL), these differences were not significant (*p* = 0.32). No differences were observed between umbilical serum osmolality in all three investigated groups (AKI 286.4 mmol/kg H_2_O, no-AKI 284.1 mmol/kg H_2_O vs controls 284.1 mmol/kg H_2_O, *p* = 0.32, respectively), and 24 h after birth (AKI 283.1 mmol/kg H_2_O, no-AKI 284.1 mmol/kg H_2_O vs controls 284.0 mmol/kg H_2_O, *p* = 0.51, respectively)—Fig. 1. However, we observed significant difference in serum osmolality between umbilical cord blood and venous blood in AKI group (*p* = 0.02).

The median serum NGAL levels in umbilical cord blood were significantly higher in the AKI group (174.3 ng/mL) compared with no-AKI (88.5 ng/mL, *p* = 0.01) and control groups (28.5 ng/mL, *p* < 0.001), and 24 h after birth (respectively AKI 152.5 ng/mL vs no-AKI 74.9 ng/mL, *p* = 0.02 vs controls 39.1 ng/mL, *p* < 0.001).

A strong negative correlation between NGAL cord blood concentration and umbilical arterial pH (Rho = −0.42, *p* = 0.04), umbilical artery base excess (Rho = −0.31, *p* = 0.03), and Apgar score in 1st min (Rho = −0.41, *p* = 0.02) and 5th min of neonatal life (Rho = −0.20, *p* = 0.001).

**Fig. 2:**
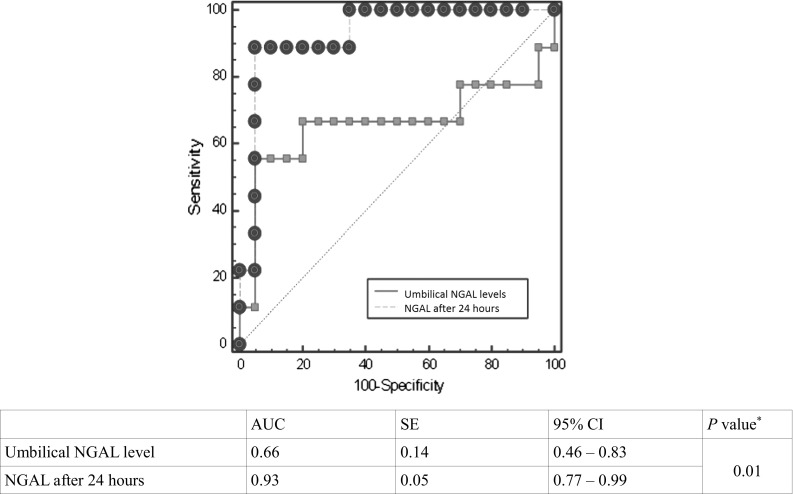
Comparison of ROC curve analysis for prediction of AKI based on umbilical and 24-h NGAL levels in asphyxiated neonates. Comparison of ROC curves (*Z* statistic = 2.14). *AUC* area under ROC curve, *SE* standard error, 95 % confidence interval for SE, *NGAL* neutrophil gelatinase-associated lipocalin, *AKI* acute kidney injury

**Fig. 3:**
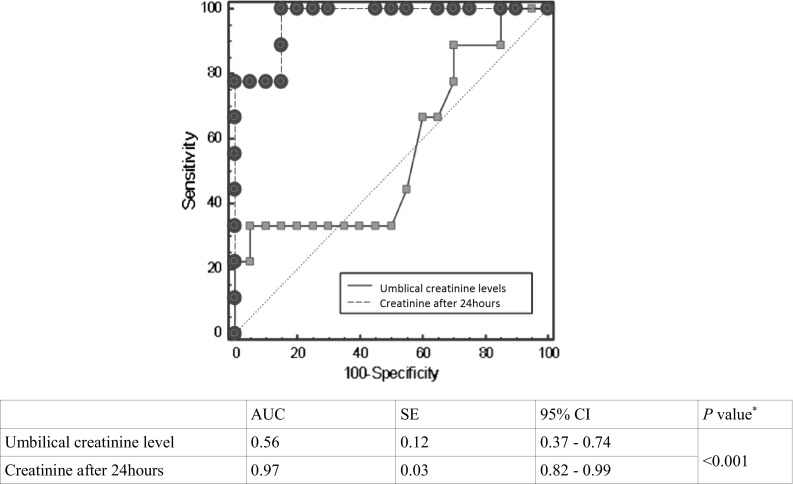
Comparison of ROC curve analysis for prediction of AKI based on umbilical and 24-h creatinine levels in asphyxiated neonates. Comparison of ROC curves (*Z* statistic = 3.50). *AUC* area under ROC curve, *SE* standard error, 95 % confidence interval for SE. *NGAL* neutrophil gelatinase-associated lipocalin, *AKI* acute kidney injury

ROC curve analysis demonstrated a critical level of NGAL in the umbilical cord blood >67.5 ng/mL, and this allows with 55.6 % sensitivity (95 % CI 28–79 %) and 89.5 % specificity (95 % CI 72–99 %) to predict AKI in newborns. However, serum NGAL concentration, 24 h after birth, with a value >140.7 ng/mL allows to diagnose AKI in asphyxiated patients with 88.9 % sensitivity (95 % CI 75–95 %) and 95.0 % specificity (95 % CI 76–99 %)—Fig. 2. On the basis of the ROC curve analysis, a critical value was established for creatinine >1.09 mg/dL in serum after 24 h in prediction of AKI in neonates with 71.4 % sensitivity (95 %CI 65–85 %) and 78.8 % specificity (95 %CI 63–90 %). However, area under ROC curve for umbilical creatinine level was not significant in prediction of AKI—Fig. 3.

## Discussion

There are many different causes of acute renal failure in newborns [1]. In this study, we have focused on asphyxiated neonates. It is very important to recognize AKI as earlier as possible, hours after an insult occurred in comparison with days it may take serum creatinine to rise. However, validation of novel AKI biomarkers is impaired by the lack of a high quality, sensitive, and specific definition of AKI in neonates [17, 18]. In umbilical cord blood, normal creatinine values are unknown; however, there are some papers about creatinine values in neonates [19].

In our study, based on AKIN criteria in 8 neonates (18.6 %) of asphyxiated group diagnosed AKI and, similarly, on the faith of neonatal AKI KIDO classification, 8 neonates developed AKI stage 1 [20].

We investigated NGAL, copeptin, creatinine levels, and serum osmolality in neonates after acute perinatal asphyxia with and without acute kidney injury, and in healthy controls.

In our studies, we determined a significant difference in NGAL concentration both in umbilical cord and venous blood after 24 h of life between asphyxiated neonates with AKI and without AKI. The ROC curve analysis revealed that serum NGAL concentration, at a cut-off value of 140.7 mg/dL after 24 h, could predict the development of AKI with high sensitivity (88.9 %) and specificity (95.0 %), while umbilical NGAL concentration characterized with low sensitivity (55.6 %), but high specificity (89.5 %) for the prediction of AKI. We suggest that the elevation of serum NGAL concentration after 24 h may play a pivotal role as an indicator of hemodynamic instability and serve as an important predictor of AKI. These data are in line with Raggal et al., who evaluated the NGAL concentration in asphyxiated neonates, 6 h after birth [21]. They demonstrated that a cut-off value of 157 ng/mL for serum NGAL could detect AKI in asphyxiated neonates with a sensitivity of 83.3 % and specificity of 94.4 %.El-Farghali et al. showed that serum NGAL may also be serve as a clinically useful marker for early detection of AKI in critically ill neonates with sepsis [22]. The kidney responds to sepsis by upregulating NGAL production [23]. NGAL is one of the most strikingly upregulated genes and is an overexpressed protein in kidneys after ischemia [18].

Our findings indicate that copeptin cannot to be a predictor of early AKI. We did not observe any significant differences of serum copeptin levels in the groups with and without AKI, as well as the control group. Serum copeptin concentrations are characterized by a low sensitivity and specificity both in umbilical cord and venous blood after 24 h. These data are, however, different than obtained by Schlapbach et al. [24]. The authors indicated the highest copeptin cord blood concentrations in neonates with perinatal asphyxia. They showed also that umbilical copeptin concentrations above 400 pmol/l had a high sensitivity and specificity for asphyxia.Copeptin pre-pro-vasopressin split product is also known as a marker of hydration status. Physiologically, when plasma osmolality is higher, copeptin levels are also increased [25]. Some authors demonstrated that in adults, during a hypertonic saline infusion and thirsting, there is a rise of serum osmolality which results in the increase in serum copeptin concentration [26].

In our study, serum copeptin concentration did not reveal significant diagnostic elements as a marker of osmolality. We have observed a significantly lower serum osmolality, in the umbilical cord than in venous blood after 24 h, in neonates with AKI, but there were no significant differences in serum copeptin concentration. This could be as a result of possible subclinical kidney injury.

A non-significant decrease in copeptin concentration 24 h after birth has also been shown in the group with no-AKI and control group, however, without any differences in serum osmolality. Due to the absence of differences in copeptin concentration in asphyxiated infants (AKI group and no-AKI) and healthy children, we suggest that copeptin cannot be a valuable, early marker of asphyxia and AKI in neonates. However, Benzing et al. disagree with this statement [27]. The authors postulated that cord blood copeptin is highly sensitive albeit minimally specific marker of fetal/neonatal distress.

Most likely the small number of investigated infants contributed to the difference between the results.

## Conclusions

Elevated serum NGAL concentration seems to be a promising, novel marker, even in subclinical forms of AKI in asphyxiated neonates, due to its high specificity, but copeptin did not meet such expectations.
